# Differentiating methicillin resistant and susceptible *Staphylococcus aureus* from ocular infections using photoacoustic labeling

**DOI:** 10.3389/fmed.2023.1017192

**Published:** 2023-02-23

**Authors:** Robert H. Edgar, Anie-Pier Samson, Regis P. Kowalski, John A. Kellum, John Hempel, John A. Viator, Vishal Jhanji

**Affiliations:** ^1^Swanson School of Engineering, Bioengineering, University of Pittsburgh, Pittsburgh, PA, United States; ^2^Department of Engineering, Duquesne University, Pittsburgh, PA, United States; ^3^School of Medicine and Ophthalmology, University of Pittsburgh, Pittsburgh, PA, United States; ^4^Center for Central Care Nephrology, Department of Critical Care Medicine, University of Pittsburgh, Pittsburgh, PA, United States; ^5^Spectral Medical, Toronto, ON, Canada

**Keywords:** flow cytometry, optoacoustics, ultrasonic, k-means, microbial

## Abstract

**Introduction:**

Antibiotic resistance in bacterial species constitutes a growing problem in the clinical management of infections. Not only does it limit therapeutic options, but application of ineffective antibiotics allows resistant species to progress prior to prescribing more effective treatment to patients. Methicillin resistance in *Staphylococcus aureus* is a major problem in clinical infections as it is the most common hospital acquired infection.

**Methods:**

We developed a photoacoustic flow cytometer using engineered bacteriophage as probes for rapid determination of methicillin resistance in *Staphylococcus aureus* with thirteen clinical samples obtained from keratitis patients. This method irradiates cells under flow with 532 nm laser light and selectively generates acoustic waves in labeled bacterial cells, thus enabling detection and enumeration of them. *Staphylococcus aureus* isolates were classified from culture isolation as either methicillin resistant or susceptible using cefoxitin disk diffusion testing. The photoacoustic method enumerates bacterial cells before and after treatment with antibiotics. Decreasing counts of bacteria after treatment indicate susceptible strains. We quantified the bacterial cells in the treated and untreated samples.

**Results:**

Using k-means clustering on the data, we achieved 100% concordance with the classification of *Staphylococcus aureus* resistance using culture.

**Discussion:**

Photoacoustics can be used to differentiate methicillin resistant and susceptible strains of bacteria from ocular infections. This method may be generalized to other bacterial species using appropriate bacteriophages and testing for resistance using other antibiotics.

## 1. Introduction

Methicillin resistant *Staphylococcus aureus* (*S. aureus*) (MRSA) infections are on the rise in both community and hospital settings (1, 2). Several studies have shown an increased rate of MRSA infections and that they account for the majority of clinically treated eye infections and they are the second most prevalent health care associated infection after *Pseudomonas aeruginosa* (3–5). Resistance to antibiotics is a natural process that has accelerated by human use of antibiotics for medicine and agriculture. In an effort to slow the rate of growth of antibiotic resistance, rapid identification and assessment of bacteria is essential so that antibiotics can be properly selected. New antibiotics have been slow to develop with only two completely novel antibiotics brought into use in the last 75 years (6). In the United States, an estimated $30 billion is spent annually on dealing with antibiotic resistant bacteria (7).

One group of bacteria is primarily responsible for the majority of multi-drug resistant infections. The ESKAPE pathogens ( *Enterococcus faecium, Staphylococcus aureus, Klebsiella pneumonia, Acinetobacter baumannii, Pseudomonas aeruginosa, and Enterobacter*) are responsible for the majority of nosocomial infections and are most likely to be multi-drug resistant (6). An estimated 90% of human antibiotic use is broad spectrum prescribed (7). From food processing plants to hospital beds, the speed at which bacterial contamination can be identified is the most important factor in treatment and control. Rapid bacterial identification negates the need for broad spectrum antibiotic use and allows for targeted therapy. Characterization of these pathogens not only requires their identification, but also determining their susceptibility to antibiotics. Ideally, rapid bacterial detection and identification of resistance would be fast enough to negate the use of broad spectrum antibiotics, therefore allowing point of care facilities to test and obviating the need for expensive equipment.

We have developed a photoacoustic method for detection of dilute particles in body fluids, *in vitro* (8, 9). Much of this work has been used for detecting circulating tumor cells (CTCs), primarily melanoma cells, as their native optical absorber, melanin, makes them suitable for sensitive photoacoustic detection and enumeration. This photoacoustic method is a type of flow cytometry in which cell suspensions are irradiated with nanosecond laser light that targets optically absorbing particles. These particles subsequently generate acoustic waves that are then detected using ultrasonic transducers. These signals are then counted, providing information about the number of particles. This number has been used to indicate disease state in cancer patients by determining the relative number of CTCs over time. Using bacteriophage that are engineered to absorb laser light, we specifically label bacterial cells in a sample and are able to perform the same type of photoacoustic flow cytometry (10–12). We showed that, like CTCs, we can detect single bacterial cells using this method.

**Figure 1:**
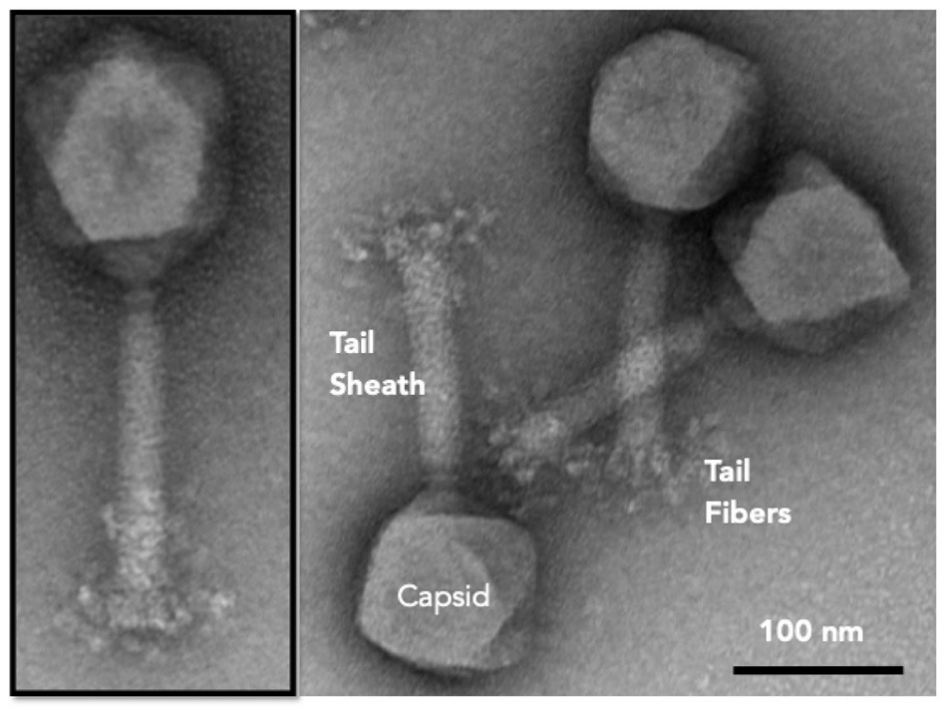
DET7 bacteriophage showing polyhedral capsid, tail sheath, and tail fibers with recognition proteins that attach to specific bacteria.

For bacterial detection under flow, we need to induce optical absorption in these otherwise colorless cells. Bacteriophage viruses have evolved to have tail fibers that specifically recognize and bind to target bacterial cells. Figure 1 shows a DET7 bacteriophage that specifically binds to 60% of the 2,700 serovars of salmonella. If one of these serovars of salmonella is present among DET7 bacteriophage, DET7 will bind to the surface and eventually infect the cell by injecting the virus's own genetic material. We add a red protein dye to the bacteriophage so that after recognizing and binding to their target bacteria, we have essentially painted the bacterial cells with red dye, allowing us to target these cells with our laser and generate ultrasonic waves in our photoacoustic flow cytometer. Conceptually, for clinical testing to identify a bacterial infection in a patient, the infected blood sample will be split into several subsamples. A different type of bacteriophage will be added to each subsample. Only the subsample with the bacterial species that matches the bacteriophage will be painted with dye and, hence, will be the only subsample that results in photoacoustic detections. This is the basic method for rapid bacterial identification.

Once the infection is identified, a new blood sample can be split into two subsamples. Presumably, each subsample will contain approximately the same number of bacteria. One sample will be treated with an antibiotic agent while the other will be untreated. After two hours, both subsamples will be tested with the photoacoustic flow cytometer which will count the bacterial cells. If the number from both subsamples is approximately the same, we can assume that the bacteria is resistant to that antibiotic. If the treated subsample has significantly fewer cells, indicated by the number of photoacoustic events, then antibiotic susceptibility is indicated.

Competing technologies using polymerase chain reaction (PCR), fluorescence in situ hybridization (FISH), or bacterial culture require amplification, either in terms of genetic material or organism number (13–17). This requirement introduces significant delays in returning results and may introduce contamination. Moreover, both PCR or FISH involve analysis of molecular material, rather than the culprit bacteria themselves. Standard blood culture and PCR are the only methods in widespread clinical use. Though relatively inexpensive, blood cultures require an incubation period of 24–96 h, with the possibility of unsuccessful culturing or of culturing a competing bacterial species that is not the cause of pathology. The cost of PCR is highly variable, on the order of $100, and still requires a culturing period, with the complication of choosing targeted reagents and amplifying unexpressed genes.

There are other research attempts for early detection of bacterial infection, including size, mechanical property, electrical, and acoustic types of classification (18–21). While these methods are innovative and exploit powerful techniques, they fundamentally work as enrichment tools, as these properties are all subject to high levels of biological variability. Consequently, discrimination must be done with higher sensitivity and lower specificity, so that most bacterial cells are selected, at the cost of excess blood cell contamination. The major shortcoming in all such methods is that they do not select for bacteria, but instead select for ranges of properties of bacterial cells that are shared with most bacterial cell types.

In this study, we obtained samples of *S. aureus* from patients who were treated for microbial keratitis. We tested each sample photoacoustically and determined whether these samples were methicillin resistant and compared our results to genetic testing for methicillin resistance.

## 2. Materials and methods

### 2.1. Photoacoustic system

**Figure 2:**
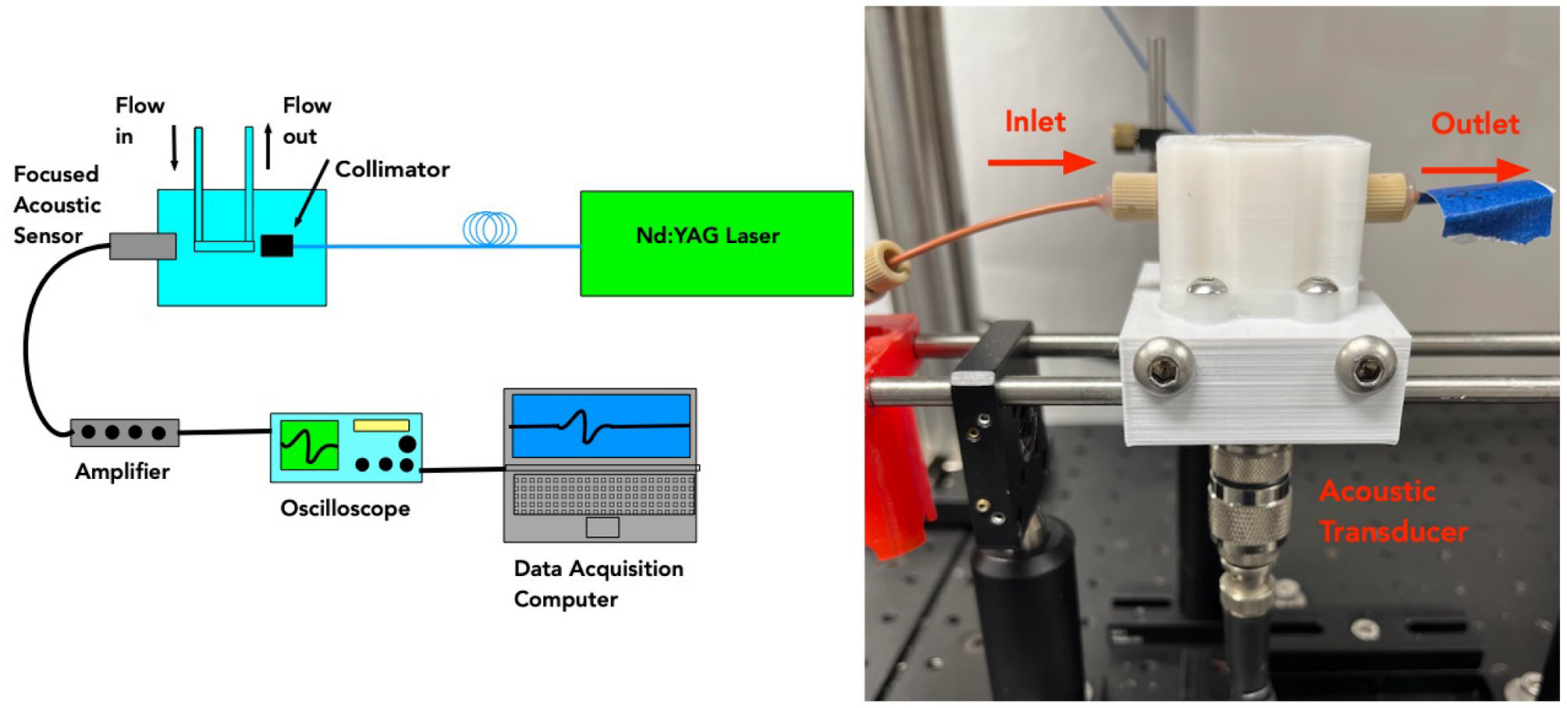
**(Left)** Schematic showing photoacoustic setup used for testing *S. aureus* samples. **(Right)** The actual flow chamber is shown.

Our photoacoustic system is shown in Figure 2 and consisted of a laser coupled to an optical fiber directed at a flow chamber through which saline suspensions were directed. An acoustic transducer was coupled to detection electronics for data processing and analysis. The system was calibrated using 10 μm dyed polystyrene microspheres (Polybead, Warrington, Pennsylvania) and phosphate buffered saline (Fisher Scientific, Pittsburgh, Pennsylvania) as positive and negative controls. As a control for bacteriophage binding, we used American Type Culture Collection strain 35556 (*S. aureus* strain SA113, ATCC, Manassas, Virginia). Modified bacteriophage SP1 was used as tags at a ratio of 1,000 bacteriophage per *S. aureus* cell. Bacteriophage were added to each culture and incubated at room temperature for 10 minutes to ensure phage binding. The combined culture and bacteriophage mixture was passed through the PAFC system at a flow rate of 60 μL/min.

**Figure 3:**
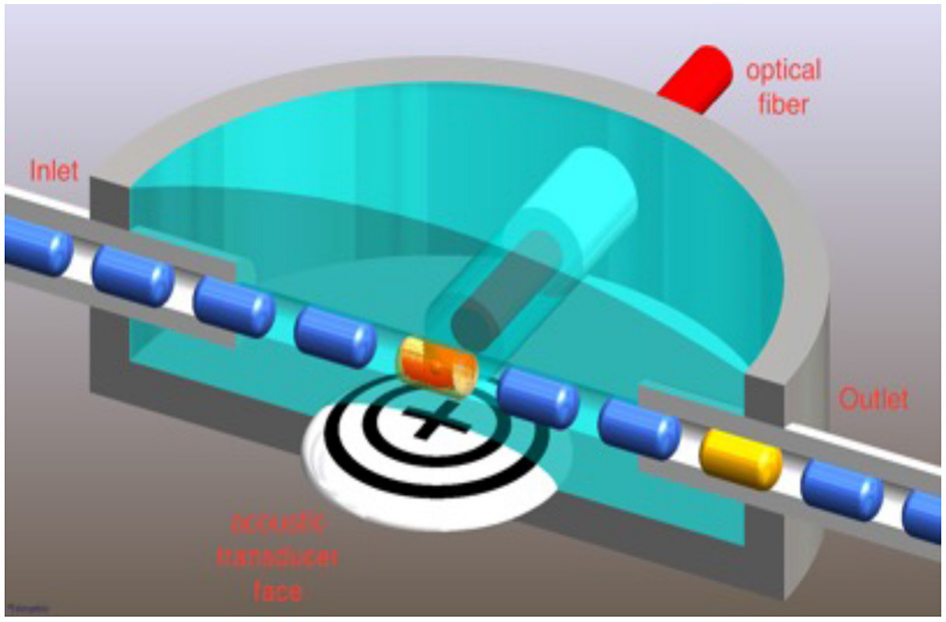
The flow chamber has alternating droplets of cell suspension and mineral oil so that detected cells are sequestered for downstream capture.

The photoacoustic flow cytometer used a frequency doubled Nd:YAGlaser operating at 532 nmwith a 5 nspulse duration and a 20 Hzpulse repetition rate. These laser parameters are appropriate for inducing acoustic waves in labeled bacteriophage attached to bacterial cells. Laser light was launched into a 1,000 μmoptical fiber with a numerical aperture of 0.22 (Thorlabs, Newton, New Jersey). The optical fiber was directed to a flow chamber made from 3D printed polylactic acid (PLA) filament. The chamber is shown in Figure 3. An immersion acoustic transducer (Olympus, Waltham, Massachusetts) fixed to the flow chamber with a center frequency of 2.25 MHzand a focal length of 0.5 inches was used to sense the generated acoustic waves.

Rather than sending a continuous flow of cell suspension through the flow chamber, we induced two phase flow by introducing an immiscible fluid to the saline suspension. We used mineral oil, thus creating alternating droplets of cell suspension and oil (22, 23). These alternating droplets created a fluidic conveyor belt that allowed for localized detection of photoacoustic events. This arrangement allowed for microfluidic capture of droplets that generated photoacoustic waves which identified bacterial cells of interest.

The transducer was coupled to a high frequency digitizer and amplifier (National Instruments, Austin, Texas) connected to a desktop computer (Dell, Round Rock, Texas). Photoacoustic waves were identified by a LabVIEW (National Instruments, Austin, Texas) program made for this photoacoustic flow cytometer. Photoacoustic events were classified by a simple threshold of the voltage signal from the transducer. The threshold was set at three times the standard deviation of the noise. Each photoacoustic wave was assumed to be generated from a single bacterial cell, which was reasonable from the dilute concentration of bacterial cells. The bacterial count was recorded for each patient sample which as split into two subsamples, one of which was treated with oxacillin, and one was untreated. These numbers were used for determination of antibiotic resistance.

For quality control, we calibrated the photoacoustic system before each use. We ran a sample of phosphate buffered saline (PBS) as a negative control. In all PBS samples, we detected no photoacoustic events, as expected, as there were no optical absorbers present. For a positive control, we ran a suspension of 1 μmblack latex microspheres to ensure we were successfully detecting photoacoustic events. In all such cases, we showed constant detections, as the microspheres generated photoacoustic waves.

### 2.2. Sample preparation

*S. aureus* samples were obtained from The Charles T. Campbell Eye Microbiology Laboratory, University of Pittsburgh Medical Center, Pittsburgh, Pennsylvania, USA. A total of 13 samples were tested for the methicillin resistance using disk diffusion (24), thus determining their methicillin resistance before photoacoustic testing. Streaks of each *S. aureus* strain were grown on mannitol salt agar plates. Single colonies from each streak plate were used to regrow strains in mannitol salt broth for 2 h in a shaking water bath at 36.5 °C. This period ensured cells were growing and entering exponential growth phase. Oxacillin was added at a final concentration of 1 μg/ mlto half of each culture and grown for an additional 2 h (25). We cultured on agar plates solely for comparison to plate reader results. The photoacoustic method, in clinical implementation, will test samples directly taken from patients without the culture phase.

Before processing through PAFC system, 100 μLfrom each culture was removed and used for growth analysis in an H1 plate reader (Biotek, Winooski, Vermont). This procedure was done to verify the photoacoustic method and were not integral to photoacoustic testing. Growth curves were made for each culture by taking the optical density of each treated and untreated culture every minute over a 16 hour period. We determined that two hours of antibiotic treatment was sufficient to determine differential growth rates from prior experimentation. Prior to performing photoacoustic testing, treated and untreated samples were incubated side-by-side for two hours. Photoacoustic testing of treated and untreated samples for each isolate were alternated, so that both samples were tested within twenty minutes to allow for similar growth times. Thus, total bacteria number could be compared.

### 2.3. Bacteriophage preparation

Bacteriophage were produced using SA113 (ATCC, Old Town Manassas, Virginia) and bacteriophage lysates were concentrated using polyethylene glycol 8000 (PEG) precipitation. Differential centrifugation and cesium chloride gradients were used to further concentrate and purify stocks to a concentration greater then 5 × 10^11^ plaque forming units per milliliter (PFU/mL). These bacteriophage concentration methods are previously described by Edgar et al., Nielson et al., and Yamamoto et al. (11, 26, 27). Bacteriophage were modified to increase absorbance of 532 nmlaser light by adding Direct Red 81 dye (Sigma Aldrich, Saint Louis, Missouri). SP1 bacteriophage were grown using SA113. Purified phage of 10^12^ plaque forming unites per milliliter (PFU/ml) were added to a saturated solution of Direct Red 81 dye. Virion particles were then pelleted and resuspended in buffer (10 mM Tris, pH 7.5, 10 mM MgCl_2_, 68 mM NaCl). This process was repeated to ensure the removal of unbound dye. The absorbance spectrum of dyed phage was determined using the H1 plate reader and compared to that of undyed phage particles. Dyed phage were titred to ensure no detrimental effects were observed from the dying process. Dyed phage were retested for their ability to infect after 150 days and no difference in titer was observed.

Efficacy of Direct Red 81 to induce photoacoustic responses in bacteriophage has been previously reported (11). We also showed that bacterial cells and bacteriophage without Direct Red 81 dye showed no photoacoustic response, even at high titer. This result is consistent with the absence of optical absorption at 532 nm, the laser excitation wavelength.

### 2.4. k-means clustering

In order to interpret the photoacoustic data provided by the flow cytometer, we used k-means clustering to guide our differentiation between methicillin resistant and susceptible samples (28). Although a simple interpretation of the number of bacteria after oxacillin treatment compared to the number in the untreated subsample should obviously indicate whether the bacteria was methicillin resistant or not, we used a formal method to automatically determine resistance. K-means, like other clustering methods, takes data points in a space of one or more dimensions and determines natural groupings of those points by proximity. Given a number of clusters, k, the algorithm separates all points into that number of groups. Since we are only interested in resistant and susceptible groups, we chose k = 2. We took the ratio of treated detection numbers over untreated numbers resulting in 13 numbers, approximately in the range of 0 to 1. Lower numbers would indicate that oxacillin was effective in decreasing the *S. aureus* population. However, there was no *ad hoc* threshold for determining antibiotic resistance. We applied k-means with two clusters to this data set. We used the Matlab function, kmeans, which uses Lloyd's algorithm. For simplicity, we used Euclidean distance for measuring and establishing iterative clusters. This analysis resulted in two clearly defined clusters for MRSA and methicillin susceptible samples.

## 3. Results

Table 1 lists each of the clinical isolates obtained from the patients. We indicate how many photoacoustic events and, hence, how many bacterial cells were detected in the oxacillin treated and untreated subsamples. Photoacoustic testing resulted in bacterial counts ranging from 2 to 689 at 2 h when incubated with oxacillin compared to 88 to 818 when samples incubated without antibiotics. Since the sample size was small and the distribution was not obviously Gaussian, we performed a nonparametric test to compare the means of the bacterial counts before and after treatment with oxacillin. Using a Wilcoxon matched pairs signed rank test, we calculated a p-value of 0.0007. Furthermore, we observed two distinct subpopulations when incubated with oxacillin. In one subgroup growth rates were similar between treated and untreated conditions, with a mean ratio of treated to untreated of 0.87, while the second group was markedly different with a mean ratio of 0.10. Once again, due to the limited sample size, we performed a nonparametric test of the two groups. Using a Mann–Whitney test, we calculated a *p*-value of 0.0012.

**Table 1:** Comparison of treated and untreated photoacoustic detections of *S. aureus*.

**Patient**	**Untreated**	**Oxacillin treated**	**k-means**	**Disk diffusion**	**Concordance:**
	**detections**	**detections**	**cluster**	**verified by disk**	**k-means and disk**
K3255	798	53	1	No	Yes
K3251	726	7	1	No	Yes
B1899	611	404	2	Yes	Yes
K3282	818	689	2	Yes	Yes
K3266	119	18	1	No	Yes
K3268	88	110	2	Yes	Yes
K3270	144	134	2	Yes	Yes
K3279	198	175	2	Yes	Yes
K3261	170	135	2	Yes	Yes
K3262	183	10	1	No	Yes
K3237	137	44	1	No	Yes
K3287	210	2	1	No	Yes
K3280	227	179	2	Yes	Yes

Seven of the 13 clinical isolates were found to be methicillin resistant using disk diffusion. Isolates found to be antibiotic resistant corresponded to the subgroup with similar growth rates. To confirm the groupings by photoacoustic detection results, we used an unbiased clustering method, k-means. The k-means column in Table 1 shows whether these numbers determined a ratio that clustered in group 1 or 2, denoting susceptible or resistant, as determined by the Matlab algorithm. The k-means algorithm resulted in 100% concordance with the known antibiotic resistance.

## 4. Discussion

*S. aureus* is a common cause of bacterial keratitis, conjunctivitis, and endophthalmitis. Given the frequency of MRSA in this population, presumptive treatment for MRSA is required until sensitivity testing can be completed. While vancomycin is often used for treatment of MRSA keratitis, it is associated with corneal toxicity (29). The clinical significance cannot be overstated, as MRSA keratitis is often part of a series of comorbidities that affect visual function. While photoacoustics can certainly identify the foundational bacterial infection and provide insight into factors that can be used to manage therapy for the infection, we are investigating ways to adapt the photoacoustic method for wider application in keratitis, which manifests in a complex environment that is still clinically challenging.

### 4.1. Importance of determination of antibiotic resistance

We have previously shown that this approach can be used to detect pathogens in blood and more broadly, the spread of MRSA is an increasing clinical problem for multiple types of infection in and out of hospital. Detection and treatment of MRSA have lagged behind the spread of infections (30). The emergence of resistant bacteria is compounded by prescription of non-targeted antibiotics. Rapid identification of bacterial infection is a pressing need in clinical care. It is only after identification of the pathological agent that virulence, antibiotic resistance, and other relevant factors can be considered when determining optimal antimicrobial therapy (7, 18). Misdiagnosis can result in delayed therapy which can lead to serious complications. For some infections, delayed treatment can result in sepsis, multiple organ failure, and even death. Evaluating patients suspected of bacterial infection is a complex process with unique aspects of each case that may confound proper diagnosis. A system that can immediately identify the bacterial pathogen will result in better outcomes for millions of patients each year. Over 1.5 million cases of sepsis occur annually in the United States alone (31). For patients enrolled in clinical trials, hospital mortality has fallen to about 20% (32). However, sepsis trials fail to recruit patients where the clinical diagnosis is missed and often exclude the most severely affected. Even so, mortality exceeds 30% at 90 days and 40% at one year. In the US alone, sepsis is estimated to cost more than $24 billion in hospitalization alone. Long term sequelae in survivors includes chronic lung disease, such as fibrosis, and end-stage kidney disease.

Another major reason to identify MRSA is that it requires treatment with vancomycin or other non-penicillin, non- cephalosporin, anti-staphylococcal agents. Intravenous use of these agents can produce major toxicity especially to the kidney and can result in renal failure (6). By contrast, methicillin susceptible infection can be treated with a variety of less toxic antibiotics including cephalosporins and thus avoid the risk of renal failure. However, determination of methicillin susceptibility vs MRSA can take up to 72 h using standard techniques, such as antibiotic disk diffusion and when the infection is not in the bloodstream, it sometimes cannot be determined at all (33, 34). For example, cultures are often negative with pneumonia.

Our photoacoustic system has the potential to identify and determine antibiotic resistance from patient samples. In this study, we determined antibiotic susceptibilities for 13 clinical isolates using bacteriophage tags and our photoacoustic flow cytometry system. Each isolate had previously been determined to be methicillin resistant or susceptible through disk diffusion testing. Additionally, we made growth curves of each isolate in the presence or absence of oxacillin to reconfirm the disk diffusion classification of resistance. In each case, 16-h growth curves confirmed the disk diffusion classification as susceptible or resistant.

### 4.2. Cluster analysis

For each clinical isolate of *S. aureus*, one would expect the number of photoacoustic detections to be significantly fewer in the antibiotic treated sample than in the untreated sample for a methicillin susceptible strain. For a methicillin resistant strain, the detections should be roughly equal. There might even be slightly more detections in the treated sample in this case due to variability in splitting the sample and in the photoacoustic system. Simple visual inspection and ad hoc classification of results differentiated susceptible and resistant strains in this manner. To further strengthen this observation, we used k-means clustering to provide an objective means for separating the set of samples into two groups, namely, resistant and susceptible strains. K-means analysis of the photoacoustic events produced two clusters that grouped perfectly with the previously determined antibiotic resistance. Although k-means clustering was consistent with the previously determined nature of the samples, in the future, we will develop a classifier, rather than a clustering method, so that we can determine resistance or susceptibility to antibiotics from single samples in the clinic.

### 4.3. Accuracy of test

In this pilot study of 13 isolates, we achieved 100% concordance with disk diffusion testing for antibiotic resistance. The disk diffusion test can be considered ground truth so that classical measures of accuracy, such as sensitivity, specificity, positive predictive value, and negative predictive value, are trivially 100%, as there are no false positive or false negative results. It would be naïve to claim that the photoacoustic method has a 100% accuracy, even though this work indicates a high level of accuracy. In order to gain some measure of the usefulness of the photoacoustic method, we used a Bayesian technique to quantify the ability to determine antibiotic resistance. If we consider this experiment as a Bernoulli trial, we can use a Beta distribution as a conjugate prior which, along with the trial information, can give a posterior distribution of the probability of determining antibiotic resistance (35).

**Figure 4:**
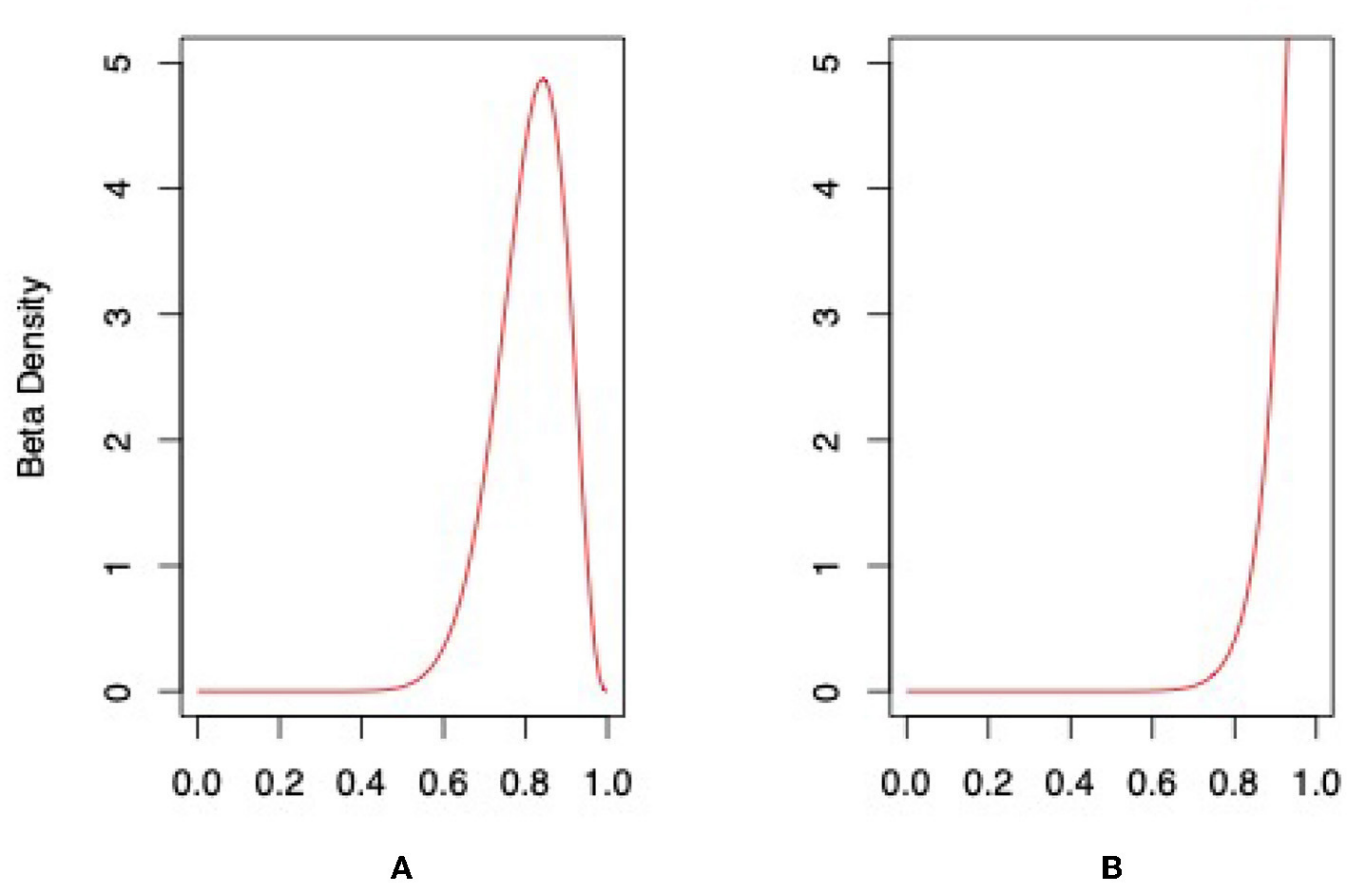
Posterior distributions for photoacoustic test using **(A)** uniform conjugate prior and **(B)** high accuracy conjugate prior.

Although choice of the conjugate prior may be arbitrary, we calculated two probability distribution based on two types of conjugate priors, (1) a uniform prior, which assumes the test has an equal chance of correctly and incorrectly determining antibiotic resistance and (2) a high accuracy prior distribution with a 0.80 probability of correctly determining antibiotic resistance. For the two Beta distributions, we used Beta(α, β) = Beta(4, 4) and Beta(5, 2). The posterior distributions are shown in Figure 4 and are Beta(17, 4) and Beta(18, 1) for the uniform and high accuracy priors, respectively. The mean and variance for the uniform distribution are 0.81 and 0.007. For the high accuracy Beta, the mean and variance are 0.95 and 0.002. This brief analysis shows that even for the unlikely assumption that the test has a uniform prior probability, the accuracy is strong, with a mean probability occurring at 0.81. The more likely assumption, based on knowledge of the 13 trials and the strength of the technology, gives a mean probability of 0.95. In any case, the Bayesian analysis gives some indication of high accuracy in the absence of false positive and negatives in the data.

## 5. Conclusions

Rapid identification and early treatment of bacterial infections has been a goal for medicine since resistant strains emerged. Control of many bacterial strains has been put into jeopardy with the rise of antibiotic resistant strains. Early detection of bacterial strains and characterization of resistance is fundamental to modern clinical treatment (31). Our method exploits the specificity of naturally derived bacteriophage probes and the robust nature of laser induced ultrasonics to provide a rapid, unambiguous method for objective identification of bacterial species and their antibiotic susceptibility.

## Data availability statement

The original contributions presented in the study are included in the article/supplementary material, further inquiries can be directed to the corresponding author.

## Author contributions

RE: experimental methods and data collection. A-PS, RK, JK, and JH: manuscript writing, reviewing, and technical improvements. JV: photoacoustic technology, data anaylsis, writing, and reviewing. VJ: clinical sample preparation and ophthalmalogy expertise. All authors contributed to the article and approved the submitted version.
